# Safety, tolerability, pharmacokinetics and pharmacodynamics of milvexian with aspirin and/or clopidogrel in healthy participants

**DOI:** 10.1038/s41598-024-67182-8

**Published:** 2024-07-18

**Authors:** Vidya Perera, Grigor Abelian, Joseph Luettgen, Ronald Aronson, Danshi Li, Zhaoqing Wang, Liping Zhang, Susan Lubin, Samira Merali, Bindu Murthy

**Affiliations:** 1 Bristol Myers Squibb, Princeton, NJ USA; 2 Janssen Research & Development, LLC, Titusville, NJ USA

**Keywords:** Thrombosis, Pharmacology

## Abstract

Milvexian, an oral activated Factor XI (FXIa) inhibitor, is in clinical studies where it may be combined with antiplatelet agents, including aspirin and/or clopidogrel, to prevent thromboembolic diseases. This phase I trial assessed safety, pharmacokinetics, and pharmacodynamics of milvexian coadministration with aspirin and/or clopidogrel in healthy participants through 3 drug-drug interaction studies using a 3-period, 3-treatment, crossover design. A total of 113 participants were randomized to receive milvexian (200 mg; twice daily for 5 days) or matched placebo coadministered with once-daily aspirin (325 mg for 5 days) and/or clopidogrel (Day 1: 300 mg; Days 2–5: 75 mg). Milvexian was safe and well tolerated, with and without aspirin and/or clopidogrel. Eight mild bleeding adverse events (AEs) were reported in 5 of 113 participants across various treatment arms. Peak and total exposures of milvexian were similar with or without clopidogrel and/or aspirin. Exposure-dependent prolongation of activated partial thromboplastin time and reduction of FXI clotting activity by milvexian were similar with coadministration of aspirin and/or clopidogrel. Milvexian, with or without coadministration of aspirin and/or clopidogrel, did not affect bleeding time or platelet aggregation. Administration of milvexian alone or with aspirin and/or clopidogrel was safe and well tolerated without increased incidence of AEs, including bleeding. Pharmacokinetic and pharmacodynamic effects of milvexian, including bleeding time, were similar with or without aspirin and/or clopidogrel.

*ClinicalTrials.gov Identifier*: NCT03698513.

## Introduction

The clinical benefits of antithrombotic therapy are well established for the prevention of thromboembolic events in patients with a broad range of existing cardiovascular diseases, such as nonvalvular atrial fibrillation, acute coronary syndrome (ACS), coronary artery disease (CAD), and peripheral artery disease (PAD)^1,2^. Although improvements in the therapeutic index of anticoagulant therapy have been made, dose-dependent bleeding continues to be observed^3^. Therefore, improving the benefit/risk profiles remains a viable goal for anticoagulant drug discovery.

Blood coagulation involves the activation of plasma proteases, their cofactors, and platelets, with 2 distinct coagulation pathways that converge at Factor X^4,5^. One coagulation pathway, the intrinsic pathway, is important in pathological conditions (ie, thrombotic and thromboembolic events), but not for hemostasis^6^. Factor XI (FXI) is a component of the intrinsic pathway and has been proposed to play an important role in maintaining and propagating a formed thrombus^7,8^. Activated FXI (FXIa) enhances the stability of clots and amplifies thrombin generation when coagulation is initiated by tissue factor or the intrinsic pathway^7,8^. Clinical, preclinical, and epidemiologic studies have shown that modulation of FXI may provide a novel mechanism for systemic anticoagulation without increasing the risk of clinically relevant bleeding in a variety of conditions associated with a high risk of thrombotic events, though clinical evidence to support the use of FXI modulation is still being generated and assessed^9–12^.

Milvexian is an oral, selective, small-molecule inhibitor of FXIa, being developed for the prevention and treatment of thromboembolic events^13^. Depending on the patient population, milvexian is expected to provide an improved benefit/risk profile as replacement or add-on to current guideline-recommended antithrombotic agents that include aspirin and/or clopidogrel^14–17^. In a phase I study of healthy participants, milvexian was safe and well tolerated at doses ≤ 500 mg daily for 14 days with no major bleeding, clinically significant bleeding events, or other serious adverse events (AEs)^18^. In a phase I study of participants with normal, mildly impaired, and moderately impaired hepatic function, a single 60-mg dose of milvexian was safe and well tolerated^19^. Additionally, milvexian was safe and well tolerated in a phase I study of participants with normal renal function, and moderate or severe renal impairment who received a single 60-mg dose^20^.

Preclinical data and clinical drug-drug interaction (DDI) of a spray-dried dispersion formulation studies have indicated that milvexian is predominantly metabolized by cytochrome P450 (CYP)–3A4 with low urine excretion (< 20%)^18,19^. In comparison, aspirin is primarily hydrolyzed by human carboxylesterase 2 in both intestine and liver to salicylic acid (active metabolite with respect to inflammation, but inactive metabolite with respect to thrombosis or hemostasis), which subsequently is bioconjugated by a number of CYP enzymes, and mainly excreted through the urine^21–23^. Clopidogrel is predominately (~ 85% of absorbed drug) first-passed hydrolyzed by human carboxylesterase 1 in the liver to clopidogrel acid (an inactive metabolite), and the rest is metabolized by CYP2C19, CYP1A2, CYP2B6, CYP2C9, and CYP3A4 to mediate the bioactivation, and mainly excreted through the urine and feces^22,24^. No pharmacokinetic (PK) interactions between milvexian, aspirin, and clopidogrel were expected^18–20,25,26^, but potential interactions were investigated as part of the drug development process. This study aimed to assess the impact of coadministration of milvexian with aspirin and/or clopidogrel on safety profiles and PK and pharmacodynamic (PD) properties to support the use of these agents in combination.

## Results

### Participant characteristics

A total of 304 subjects were screened, and 113 (37.2%) subjects, all of whom were clopidogrel responders, were randomized to Parts 1 (n = 37), 2 (n = 37), and 3 (n = 39). Across groups, participants had similar baseline characteristics, including mean age, weight, and body mass index (BMI; Table 1). The mean age ranged from 33.4 to 35.1 years, 92.3% to 97.3% were male, and mean BMI ranged from 26.1 to 27.2 kg/m^2^ among participants in Parts 1 to 3 of the study. In Part 1, 35 of 37 (94.6%) randomized participants completed the study; 2 (5.4%) participants requested to discontinue treatment (not due to AEs). In Part 2, 33 of 37 (89.2%) randomized participants completed the study; 4 (10.8%) participants discontinued the study (1 due to an AE of hypotension and 3 due to the participants’ request to discontinue treatment). In Part 3, 35 of 39 (89.7%) randomized participants completed the study; 4 (10.3%) participants discontinued the study (1 due to a bleeding AE of anal fissure hemorrhage and 3 due to poor/noncompliance).

**Table 1:** Baseline Characteristics.

Characteristic	Part 1 milvexian* + aspirin^†^ + clopidogrel^‡^ (n = 37)	Part 2 milvexian* + clopidogrel^‡^ (n = 37)	Part 3 milvexian* + aspirin^†^ (n = 39)
Age, years, mean ± SD	33.8 ± 8.7	33.4 ± 10.4	35.1 ± 8.4
Sex, n (%)			
Male	36 (97.3)	35 (94.6)	36 (92.3)
Female	1 (2.7)	2 (5.4)	3 (7.7)
Race, n (%)			
White	17 (45.9)	16 (43.2)	21 (53.8)
Black or African American	16 (43.2)	18 (48.6)	13 (33.3)
American Indian or Alaska Native	0	1 (2.7)	4 (10.3)
Asian	2 (5.4)	1 (2.7)	1 (2.6)
Other	2 (5.4)	1 (2.7)	0
Weight, kg, mean ± SD	80.0 ± 12.8	79.4 ± 11.5	83.8 ± 11.8
BMI, kg/m^2^, mean ± SD	26.2 ± 3.4	26.1 ± 3.0	27.2 ± 3.0

BID, twice daily; BMI, body mass index; QD, once daily; SD, standard deviation.

*Milvexian 200 mg BID on Days 1 to 5.

^†^Aspirin 325 mg QD on Days 1 to 5.

^‡^Clopidogrel 300 mg QD on Day 1 then 75 mg QD on Days 2 to 5.

### Safety and tolerability

Milvexian 200 mg twice daily (BID) given for 5 days with and without antiplatelet therapy (clopidogrel 300 mg once daily [QD] on Day 1 then 75 mg QD on Days 2–4 and/or aspirin 325 mg QD) was safe and well tolerated. No deaths or serious AEs occurred during the study (Table 2). Mild bleeding AEs assessed as related to various treatment groups were reported in 5 participants, and no numerical imbalance in the incidence of bleeding was observed when milvexian was coadministered with dual antiplatelet therapy or with aspirin or clopidogrel, separately. Specifically, in Part 1, a single participant reported 2 events of contusion after administration of milvexian + aspirin + clopidogrel (Treatment A) and 1 event after placebo + aspirin + clopidogrel (Treatment C), and 1 participant reported 1 event of gingival bleeding and 2 events of vessel puncture–site bruise after administration of placebo + aspirin + clopidogrel (Treatment C). In Part 2, a single participant reported 1 event of contusion after administration of placebo + clopidogrel (Treatment E). In Part 3, a single participant reported 1 event of anal fissure hemorrhage after administration of milvexian alone (Treatment G), and 1 participant reported 1 event of epistaxis after administration of placebo + aspirin (Treatment H). No notable changes in electrocardiogram (ECG), vital signs, or physical examination results were observed during the study (Supplementary Tables 1–3).

**Table 2:** Summary Safety and AEs by Treatment.

	Part 1			Part 2			Part 3		
AE	Milvexian* + aspirin^†^ + clopidogrel^‡^ (Treatment A) (n = 37)	Milvexian* (Treatment B) (n = 36)	Placebo + aspirin^†^ + clopidogrel^‡^ (Treatment C) (n = 36)	Milvexian* (Treatment D) (n = 36)	Placebo + clopidogrel^‡^ (Treatment E) (n = 36)	Milvexian* + clopidogrel^‡^ (Treatment F) (n = 35)	Milvexian* (Treatment G) (n = 37)	Placebo + aspirin^†^ (Treatment H) (n = 38)	Milvexian* + aspirin^†^ (Treatment I) (n = 35)
Total participants with an AE, n (%)	5 (13.5)	5 (13.9)	7 (19.4)	5 (13.9)	6 (16.7)	5 (14.3)	6 (16.2)	9 (23.7)	5 (14.3)
Deaths, n (%)	0	0	0	0	0	0	0	0	0
Serious AEs, n (%)	0	0	0	0	0	0	0	0	0
Discontinuations due to AEs, n (%)	0	0	0	1 (2.8)^§^	1 (2.8)^‖^	0	1 (2.7)^¶^	0	0
Total participants with bleeding event(s), n (%)	1 (2.7)	0	2 (5.6)	0	1 (2.8)	0	1 (2.7)	1 (2.6)	0
Gingival bleeding	0	0	1 (2.8)^#^	0	0	0	0	0	0
Vessel puncture site bruise	0	0	1 (2.8)^#^	0	0	0	0	0	0
Contusion	1 (2.7)**	0	1 (2.8)**	0	1 (2.8)^††^	0	0	0	0
Anal fissure hemorrhage	0	0	0	0	0	0	1 (2.7)	0	0
Epistaxis	0	0	0	0	0	0	0	1 (2.6)^‡‡^	0

AE, adverse event; BID, twice daily; QD, once daily.

*Milvexian 200 mg BID on Days 1 to 5.

^†^Aspirin 325 mg QD on Days 1 to 5.

^‡^Clopidogrel 300 mg QD on Day 1 then 75 mg QD on Days 2 to 5.

^§^Participant discontinued study treatment due to a nonrelated AE of pulpitis dental after milvexian.

^‖^Participant discontinued study treatment due to a nonrelated AE of hypotension after placebo + clopidogrel.

^¶^Participant discontinued study treatment due to a related bleeding AE of anal fissure hemorrhage after milvexian.

^#^Participant reported gingival bleeding and 2 events of vessel puncture–site bruise after placebo + aspirin + clopidogrel.

**1 participant reported 2 events of contusion after milvexian + aspirin + clopidogrel in Period 1 and 1 event after placebo + aspirin + clopidogrel.

^††^Participant reported contusion after placebo + clopidogrel.

^‡‡^Participant reported epistaxis after placebo + aspirin.

### Pharmacokinetics

#### Impact of aspirin and/or clopidogrel on milvexian

**Figure 1:**
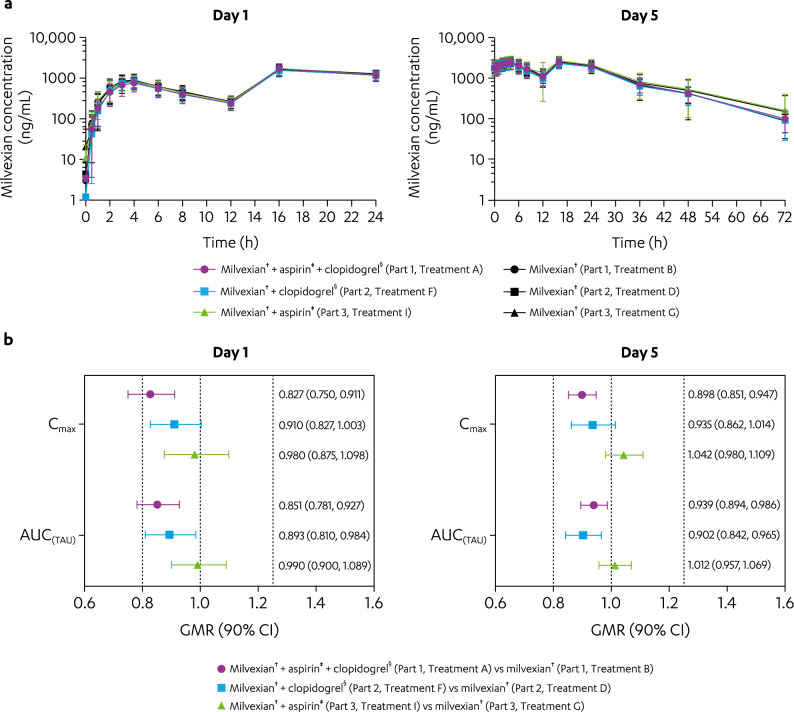
Effect of aspirin and/or clopidogrel on milvexian PK parameters on Days 1 and 5. (**a**) Mean (± SD) milvexian plasma concentration versus time profile.^*^ (**b**) GMR (90% CI) of milvexian C_max_ and AUC_(TAU)_. AUC_(TAU)_, area under the plasma concentration–time curve from time 0 to time of last quantifiable concentration; BID, twice daily; CI, confidence interval; C_max_, maximum observed concentration; GMR, geometric mean ratio; PK, pharmacokinetics; QD, once daily; SD, standard deviation. ^*^Individual plots of the curves in panel a on Days 1 and 5 can be found in Supplementary Fig. 1. ^†^Milvexian 200 mg BID on Days 1 to 5. ^‡^Aspirin 325 mg QD on Days 1 to 5. ^§^Clopidogrel 300 mg QD on Day 1 then 75 mg QD on Days 2 to 5.

After coadministration of aspirin and clopidogrel with milvexian (Treatment A), the maximum observed plasma concentration (C_max_) and area under the plasma concentration–time curve from time 0 to time of the last quantifiable concentration (AUC_(TAU)_) of milvexian were reduced by 17% (geometric mean ratio [GMR; 90% confidence interval (CI)]: 0.827 [0.750, 0.911]) and 15% (GMR [90% CI]: 0.851 [0.781, 0.927]), respectively, on Day 1, but were not changed on Day 5 compared with milvexian administered alone (Treatment B; Fig. 1). The time of maximum observed concentration (T_max_) and elimination half-life (T_1/2_) of milvexian coadministered with aspirin and clopidogrel (Treatment A) were similar to those after milvexian was administered alone (Treatment B; Supplementary Table 4). Mean peak concentration of milvexian across Treatments A and B occurred at a median T_max_ of 3 to 4 h, and mean (standard deviation [SD]) T_1/2_ after the final dose of milvexian was 12.2 (2.46) and 11.4 (1.99) h, respectively.

After coadministration of clopidogrel with milvexian (Treatment F), the C_max_, AUC_(TAU),_ T_max_ and T_1/2_ of milvexian were similar compared with milvexian administered alone (Treatment D; Fig. 1, Supplementary Table 4). After coadministration of aspirin with milvexian (Treatment I), the C_max_, AUC_(TAU),_ T_max_, and T_1/2_ of milvexian were also similar compared with milvexian administered alone (Treatment G).

#### Impact of milvexian on clopidogrel

**Figure 2:**
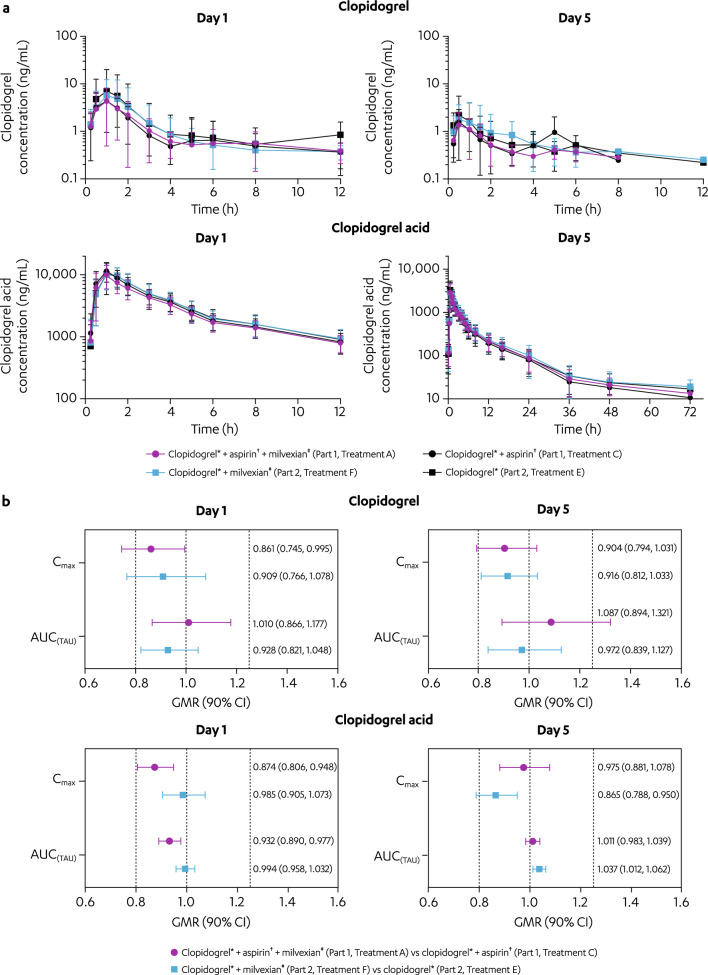
Effect of milvexian on the PK parameters of clopidogrel and its metabolite (clopidogrel acid) on Days 1 and 5. (**a**) Mean (± SD) clopidogrel and clopidogrel acid plasma concentration versus time profile*s*. (**b**) GMR (90% CI) of clopidogrel and clopidogrel acid C_max_ and AUC_(TAU)_. AUC _(TAU)_, area under the plasma concentration–time curve from time 0 to time of last quantifiable concentration; BID, twice daily; CI, confidence interval; C_max_, maximum observed concentration; GMR, geometric mean ratio; PK, pharmacokinetics; QD, once daily; SD, standard deviation. ^*^Clopidogrel 300 mg QD on Day 1 then 75 mg QD on Days 2 to 5. ^†^Aspirin 325 mg QD on Days 1 to 5. ^‡^Milvexian 200 mg BID on Days 1 to 5.

After coadministration of clopidogrel with milvexian (Treatment F), the C_max_ of clopidogrel was reduced 9.1% (GMR [90% CI]: 0.909 [0.766, 1.078]) on Day 1 but was similar compared with treatment with clopidogrel alone (Treatment E) on Day 5; AUC_(TAU)_ of clopidogrel with milvexian (Treatment F) on Days 1 and 5 were similar compared with clopidogrel alone (Treatment E; Fig. 2a and b). The C_max_ of clopidogrel acid after Treatment F was similar compared with clopidogrel alone (Treatment E) on Day 1, but reduced 14% (GMR [90% CI]: 0.865 [0.788, 0.950]) on Day 5; AUC_(TAU)_ of clopidogrel acid after Treatment F on Days 1 and 5 were similar compared with clopidogrel alone (Treatment E). When clopidogrel was coadministered with milvexian (Treatment F), median T_max_ and mean T_1/2_ of both clopidogrel and clopidogrel acid were generally comparable to the corresponding values observed with clopidogrel alone (Treatment E; Supplementary Table 5). Median T_max_ ranged from 1 to 1.5 h on Day 1 and 0.5 to 1 h on Day 5. Mean T_1/2_ values of approximately 1 to 2 h were observed for clopidogrel administered with (Treatment F) and without (Treatment E) milvexian on Days 1 and 5. Mean T_1/2_ values for clopidogrel acid were approximately 8 h in both treatments (Treatments F and E) on Days 1 and 5.

Under additional influence by aspirin (coadministration of aspirin and clopidogrel with milvexian; Treatment A), the C_max_ of clopidogrel was reduced 14% (GMR [90% CI]: 0.861 [0.745, 0.995]) and 10% (GMR [90% CI]: 0.904 [0.794, 1.031]) on Days 1 and 5, respectively, while AUC_(TAU)_ was similar on Day 1, but increased 8.7% (GMR [90% CI]: 1.087 [0.894, 1.321]) on Day 5 compared with aspirin and clopidogrel without milvexian (Treatment C; Fig. 2); the C_max_ and AUC_(TAU)_ of clopidogrel acid after Treatment A were similar on Days 1 and 5 compared with aspirin and clopidogrel without milvexian (Treatment C). When clopidogrel was coadministered with aspirin and milvexian (Treatment A), median T_max_ and mean T_1/2_ of both clopidogrel and clopidogrel acid were generally comparable to the corresponding values observed with aspirin and clopidogrel without milvexian (Treatment C; Supplementary Table 5).

#### Impact of milvexian on aspirin

**Figure 3:**
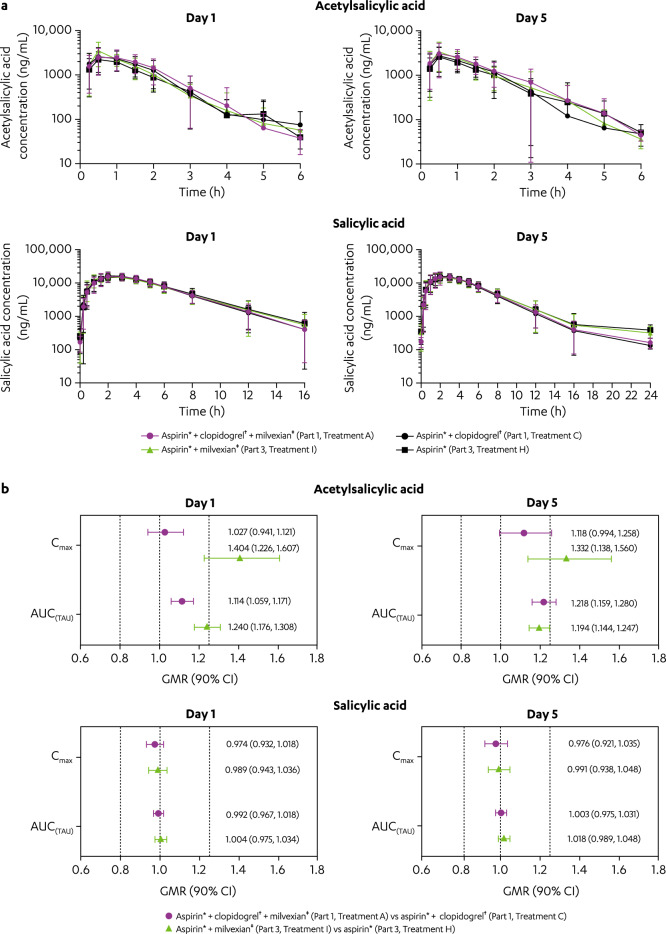
Effect of milvexian on the PK parameters of aspirin (acetylsalicylic acid) and its metabolite (salicylic acid) on Days 1 and 5. (**a**) Mean (± SD) acetylsalicylic acid and salicylic acid plasma concentration versus time profiles. (**b**) GMR (90% CI) of acetylsalicylic acid and salicylic acid C_max_ and AUC_(TAU)_. AUC _(TAU)_, area under the plasma concentration–time curve from time 0 to time of last quantifiable concentration; BID, twice daily; CI, confidence interval; C_max_, maximum observed concentration; GMR, geometric mean ratio; PK, pharmacokinetics; QD, once daily; SD, standard deviation. ^*^Aspirin 325 mg QD on Days 1 to 5. ^†^Clopidogrel 300 mg QD on Day 1 then 75 mg QD on Days 2 to 5. ^‡^Milvexian 200 mg BID on Days 1 to 5.

After coadministration of aspirin with milvexian (Treatment I), C_max_ and AUC_(TAU)_ of acetylsalicylic acid increased 40% (GMR [90% CI]: 1.404 [1.226, 1.607]) and 24% (GMR [90% CI]: 1.240 [1.176, 1.308]), respectively, on Day 1 and increased 33% (GMR [90% CI]: 1.332 [1.138, 1.560]) and 19% (GMR [90% CI]: 1.194 [1.144, 1.247]), respectively, on Day 5 compared with administration of aspirin alone (Treatment H; Fig. 3). When aspirin was coadministered with milvexian (Treatment I), the C_max_ and AUC_(TAU)_ of salicylic acid were similar on both Days 1 and 5 compared with aspirin alone (Treatment H); median T_max_ and mean T_1/2_ of both acetylsalicylic acid and salicylic acid (after Treatment I) were generally comparable to the corresponding values observed with aspirin alone (Treatment H; Supplementary Table 6).

Under additional influence by clopidogrel (coadministration of aspirin and clopidogrel with milvexian; Treatment A), the C_max_, AUC_(TAU)_, T_max_, and mean T_1/2_ of acetylsalicylic acid on Day 1 were similar compared with aspirin and clopidogrel without milvexian (Treatment C; Fig. 3); the C_max_ and AUC_(TAU)_ of acetylsalicylic acid increased 12% (GMR [90% CI]: 1.118 [0.994, 1.258]) and 22% (GMR [90% CI]: 1.218 [1.159, 1.280]), respectively, on Day 5. When aspirin and clopidogrel were coadministered with milvexian (Treatment A), the C_max_, AUC_(TAU)_, T_max_, and mean T_1/2_ of salicylic acid were similar on both Days 1 and 5 compared with aspirin and clopidogrel without milvexian (Treatment C; Supplementary Table 6).

### Pharmacodynamics

#### Impact of aspirin and/or clopidogrel on milvexian aPTT and FXIc

**Figure 4:**
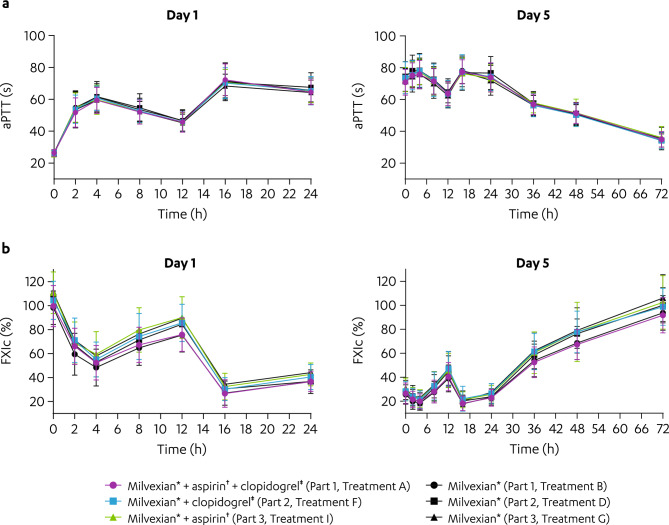
Effect of aspirin and/or clopidogrel on the PD parameters of milvexian. Mean (± SD) (**a**) aPTT and (**b**) FXIc versus time profiles of milvexian with or without aspirin and/or clopidogrel. aPTT, activated partial thromboplastin time; BID, twice daily; FXIc, Factor XI clotting activity; PD, pharmacodynamic; QD, once daily; SD, standard deviation. ^*^Milvexian 200 mg BID on Days 1 to 5. ^†^Aspirin 325 mg QD on Days 1 to 5. ^‡^Clopidogrel 300 mg QD on Day 1 then 75 mg QD on Days 2 to 5.

Exposure-dependent prolongation of activated partial thromboplastin time (aPTT) and reduction of Factor XI clotting activity (FXIc) were observed with milvexian administration, and effects were similar when milvexian was administered alone (Treatments B, D, and G) or in combination with aspirin and/or clopidogrel (Treatments A, F, and I; Fig. 4a, b). The maximum mean aPTT value ranged from 68.3 to 72.2 s at 16 h following milvexian administration alone (Treatments B, D, and G) or in combination with aspirin and/or clopidogrel (Treatments A, F, and I) on Day 1 and returned to baseline by 72 h postdose on Day 5 (Fig. 4a). Similarly, the minimum mean FXIc ranged from 26.7% to 32.5% at 16 h following milvexian administration alone (Treatments B, D, and G) or with aspirin and/or clopidogrel (Treatments A, F, and I) on Day 1 and returned to baseline by 72 h postdose on Day 5 (Fig. 4b).

#### Impact of milvexian on aspirin and/or clopidogrel on bleeding time and platelet aggregation

**Figure 5:**
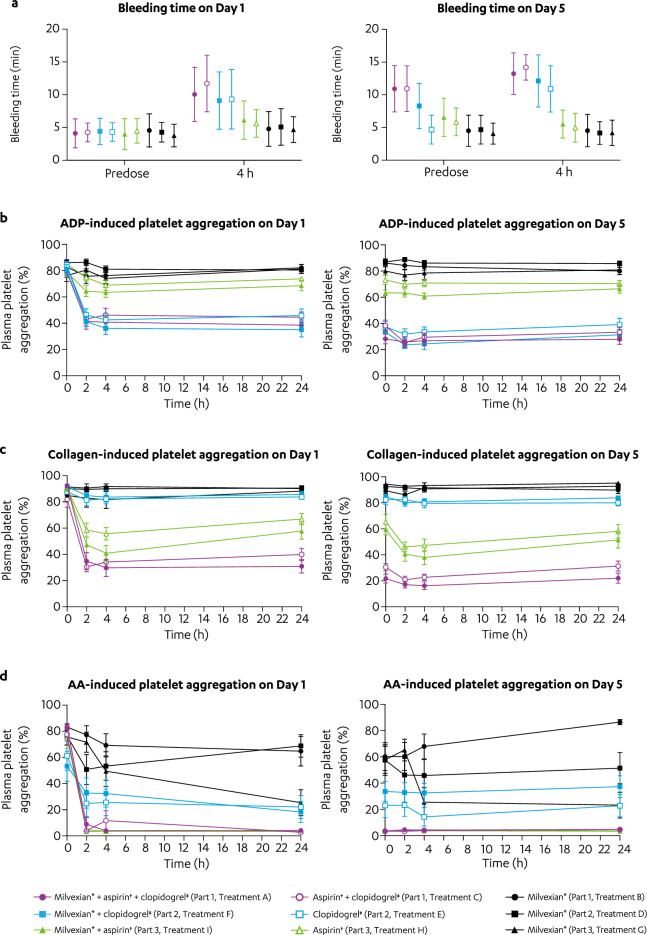
Effect of milvexian on the PD parameters of aspirin and/or clopidogrel.^*^ (**a**) Mean (± SD) bleeding time versus time profiles. (**b**–**d**) mean (± SE) plasma platelet aggregation induced by (**b**) ADP, (**c**) collagen, and (**d**) AA versus time profiles. AA, arachidonic acid; ADP, adenosine diphosphate; BID, twice daily; PD, pharmacodynamic; QD, once daily; SD, standard deviation; SE, standard of error. ^*^Milvexian 200 mg BID on Days 1 to 5. ^†^Aspirin 325 mg QD on Days 1 to 5. ^‡^Clopidogrel 300 mg QD on Day 1 then 75 mg QD on Days 2 to 5.

Mean bleeding time at baseline ranged from 3.78 to 4.56 min across treatment groups. Administration of milvexian alone (Treatments B, D, and G) did not increase bleeding times; mean bleeding times ranged from 4.16 to 5.08 min at 4 h following milvexian administration on Days 1 and 5 (Fig. 5a). Coadministration of aspirin and/or clopidogrel with milvexian (Treatments A, F, and I) increased bleeding time to a similar value as administration of aspirin and/or clopidogrel without milvexian (Treatments C, E, and H), respectively. Mean bleeding times ranged from 4.97 to 6.13 min at 4 h following administration of aspirin alone (Treatment H) or with milvexian (Treatment I) on Days 1 and 5, whereas mean bleeding times ranged from 9.1 to 12.13 min at 4 h following administration of clopidogrel alone (Treatment E) or with milvexian (Treatment F) on Days 1 and 5.

Platelet aggregation induced by adenosine diphosphate (ADP) or collagen was changed by < 10% compared with baseline following administration of milvexian alone (Treatment B, D, and G) on Days 1 and 5 (Fig. 5b, c). Although a decrease in arachidonic acid (AA)-induced platelet aggregation was observed following administration of milvexian alone (Treatment B, D, and G), this was not consistent and did not correlate with milvexian concentration (Fig. 5d). Coadministration of aspirin and clopidogrel with or without milvexian (Treatments A and C, respectively) resulted in ~ 50%, 60%, and 95% inhibition of ADP-, collagen-, and AA-induced aggregation, respectively, compared with baseline. Administration of clopidogrel alone (Treatment E) resulted in ~ 50% inhibition of ADP-induced aggregation and ~ 60% inhibition of AA-induced aggregation with little effect (< 10%) on collagen-induced aggregation compared with baseline. Administration of aspirin alone (Treatment H) resulted in ~ 15% inhibition of ADP-induced aggregation, ~ 25% inhibition of collagen-induced platelet aggregation, and ~ 95% inhibition of AA-induced platelet aggregation compared with baseline.

## Discussion

Despite clear evidence that the combination of anticoagulant and antiplatelet therapy reduces the incidence of thrombotic events in patients with cardiovascular disease (eg, ACS, CAD, or PAD), it is also associated with an increase in the risk of bleeding-related AEs^27–30^. Therefore, it is important to fully characterize the potential DDI of new anticoagulant agents with the current guideline-recommended antiplatelet drugs in these patient populations. Aspirin and clopidogrel are some of the most commonly recommended antiplatelet therapies for treatment of and to reduce the risk of thrombotic events in patients with coronary disease and non-cardioembolic stroke^14–17^. This phase I study evaluated the potential DDI between milvexian and aspirin and/or clopidogrel in healthy participants. The findings demonstrate that there is no evidence of increased risk of serious or non-serious AEs, including serious bleeding events, associated with the administration of multiple doses of milvexian with dual antiplatelet therapy (aspirin and clopidogrel) or single antiplatelet therapy (aspirin or clopidogrel) or significant changes in PK parameters of milvexian in healthy participants.

The percentage of participants reporting AEs was generally consistent across treatments in each part of the study with a slightly higher number of AEs reported after coadministration of placebo and aspirin in Part 3 (23.7% versus 13.5% to 19.4% across all other treatments). Further, the number of participants reporting bleeding AEs was similar across treatments; there was no numerical imbalance in the incidence of bleeding AEs and all bleeding AEs were mild. The results showed no evidence of an increased risk of AEs overall, including bleeding, associated with the administration of multiple doses of milvexian, with or without dual antiplatelet therapy.

Regarding PK, coadministration of aspirin and/or clopidogrel with milvexian did not affect the median T_max_ and mean T_1/2_ of milvexian, aspirin (acetylsalicylic acid) and its metabolite (salicylic acid), and clopidogrel and its metabolite (clopidogrel acid). Coadministration of aspirin and clopidogrel with milvexian did not alter the C_max_ and AUC_(TAU)_ of milvexian on Day 5, although slight reductions were observed on Day 1 (C_max_, 17%; AUC_[TAU]_, 15%; both were not statistically significant), compared with milvexian administered alone. Coadministration of milvexian with clopidogrel did not alter the C_max_ and AUC_(TAU)_ of clopidogrel and its metabolite. However, coadministration of milvexian with aspirin increased the C_max_ and AUC_(TAU)_ of acetylsalicylic acid on Day 5, compared with treatment with aspirin alone, whereas coadministration of milvexian with aspirin had no effect on the salicylic acid metabolite. Potential explanations for increased exposure in acetylsalicylic acid include: an increase in bioavailability due to disintegration of a tablet formulation of aspirin^31^, period effect, and gastric emptying rates^32,33^. Nevertheless, it should be noted that this increase in acetylsalicylic acid exposure was not expected to be clinically relevant by investigators given the small magnitude of increase.

Prolongation of aPTT and reduction of FXIc were observed after administration of milvexian with greater effects observed at higher concentrations. These observations are consistent with milvexian’s mechanism of action and previous observations in phase I studies^18–20^ and the in vitro studies and in vivo evaluation in experimental thrombosis in rabbits^34^. No additional prolongation of aPTT or reduction in FXIc were observed when milvexian was coadministered with aspirin or clopidogrel compared with administration of milvexian alone; this lack of effect on coagulation tests is expected based on the mechanism of action of clopidogrel and aspirin observed in other studies^2,21,35^. Additionally, these results are similar to previous observation from a phase I study of SHR2285 (ClinicalTrials.gov Identifier: NCT04945616), a small-molecule FXIa inhibitor, which showed that when combined with aspirin, clopidogrel, or ticagrelor, SHR2285 was safe and well tolerated with significant effects on aPTT and FXI activity and no evidence of an increased risk of bleeding^36^. Furthermore, the current study found no effects on platelet aggregation beyond the effects of aspirin and/or clopidogrel alone with coadministration of milvexian. Platelet aggregation was not affected by administration of milvexian alone. These observations are consistent with the mechanism of action of milvexian, which selectively inhibits FXIa and acts as an anticoagulant^13,18,19,34^.

Milvexian has been investigated in 2 phase II studies. In the AXIOMATIC-TKR study of patients who underwent knee arthroplasty, postoperative treatment with milvexian reduced the incidence of venous thromboembolism in a dose-dependent manner (ranging from 25 mg QD to 200 mg BID) and was associated with a low risk of bleeding^37^. The safety and efficacy of milvexian were also investigated in the phase II AXIOMATIC-SSP study of patients with acute ischemic stroke or high risk transient ischemic attack and high risk of recurrent stroke on a background of aspirin and clopidogrel^38^; milvexian (25 mg QD or 25, 50, 100, 200 mg BID) did not produce a statistically significant dose response for the composite outcome of symptomatic ischemic stroke or covert brain infarction and did not increase symptomatic intracranial or fatal bleeding compared with placebo^39^.

The current study showed no evidence of a safety impact with no increased risk of bleeding and limited impact on PK and PD profiles. A key strength of this study is that overall it is a relatively large, comprehensive phase I study design controlling for intra-individual variability across all potential interactions among the aforementioned drugs. A potential limitation of the study is the small sample sizes in each part of the study. Additional measurements of the concentrations of aspirin and/or clopidogrel and their metabolites are warranted for future studies to confirm potential DDIs of milvexian with aspirin and/or clopidogrel as well as to confirm the safety findings of the current study. Another potential limitation is the generalizability of the study findings as the study population included healthy individuals who were relatively young, thus, these results may not fully extend to patients with cardiovascular diseases or individuals of older age.

## Conclusions

This phase I DDI study in healthy adults demonstrated that administration of milvexian alone or in combination with aspirin and/or clopidogrel was safe and well tolerated and was not associated with an increased incidence of AEs, including bleeding. PK parameters and PD effects of milvexian were similar when milvexian was administered alone or in combination with aspirin and/or clopidogrel. Likewise, PK parameters and PD effects of aspirin and/or clopidogrel were generally similar when aspirin and/or clopidogrel were administered alone or in combination with milvexian. Lack of effect of milvexian on bleeding time may have promising safety implications for its potential use as an add-on to aspirin and/or clopidogrel. The results obtained in this study will help to inform the future clinical development of milvexian.

## Methods

### Participants

Healthy participants aged 18 to 55 years with a BMI of 18.0 to 32.0 kg/m^2^ were eligible for inclusion in the study. Women who were of childbearing potential or breastfeeding, or participants who had any significant acute or chronic medical illness, any other condition listed as a contraindication in the aspirin package insert, evidence of coagulopathy, or a history of bleeding were excluded from the study.

### Study design

**Figure 6:**
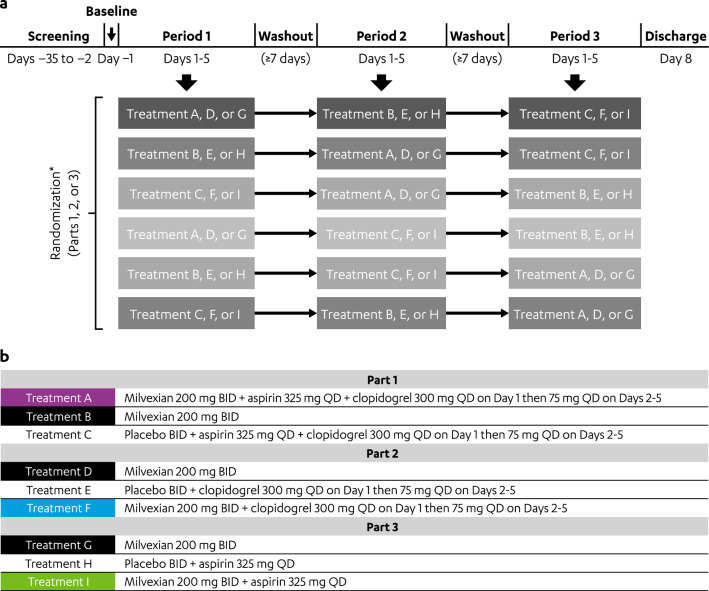
DDI study design. (**a**) A 3-period, 3-treatment, crossover study design schematic. (**b**) Dose regimens of milvexian, aspirin, and clopidogrel in Parts 1 to 3. BID, twice daily; DDI, drug-drug interaction; QD, once daily. ^*^Randomization to a treatment sequence (Parts 1, 2, or 3) occurs on Day 1.

The study (ClinicalTrials.gov Identifier: NCT03698513) was conducted at 1 clinical research center in the United States. The study planned to enroll 108 healthy participants and consisted of 3 parts; each part was a single DDI study using a randomized, 3-period, 3-treatment, crossover design (Fig. 6a and b). The following DDIs were analyzed in each part: Part 1: aspirin, clopidogrel, and milvexian; Part 2: clopidogrel and milvexian; and Part 3: aspirin and milvexian. Participants underwent screening evaluations to determine eligibility within 35 days of study drug administration, and eligible participants were randomly assigned 1:1:1 to a single part of the study. Participants randomized to Parts 1 and 2 underwent prerandomization screening to confirm that they were clopidogrel responders (ie, a decrease in platelet aggregation of ≥ 30% after a single 600-mg dose of clopidogrel compared with baseline^40^). In Part 1, randomized participants received either coadministration of milvexian 200 mg BID on Days 1 to 5, aspirin 325 mg QD on Days 1 to 5, and clopidogrel 300 mg QD on Day 1 then 75 mg QD on Days 2 to 5 (Treatment A); milvexian 200 mg BID alone on Days 1 to 5 (Treatment B); or coadministration of matched placebo BID on Days 1 to 5, aspirin 325 mg QD on Days 1 to 5, and clopidogrel 300 mg QD on Day 1 then 75 mg QD on Days 2 to 5 (Treatment C; Fig. 6b). In Part 2, randomized participants received either milvexian 200 mg BID alone on Days 1 to 5 (Treatment D); coadministration of matched placebo on Days 1 to 5 and clopidogrel 300 mg QD on Day 1 then 75 mg QD on Days 2 to 5 (Treatment E); or coadministration of milvexian 200 mg BID on Days 1 to 5 and clopidogrel 300 mg QD on Day 1 then 75 mg QD on Days 2 to 5 (Treatment F). In Part 3, randomized participants received either milvexian 200 mg BID alone on Days 1 to 5 (Treatment G); coadministration of matched placebo BID and aspirin 325 mg QD on Days 1 to 5 (Treatment H); or coadministration of milvexian 200 mg BID and aspirin 325 mg QD on Days 1 to 5 (Treatment I).

The 200-mg BID dose regimen of milvexian was selected for this study as it was shown to be generally safe and well tolerated^18,41^, and the steady-state plasma concentrations of milvexian produced by this dose were within the therapeutic ranges observed in preclinical studies^34^. Milvexian 200 mg BID was also the highest dose of milvexian planned for investigation in phase II studies. Aspirin 325-mg tablets were investigator-sourced in commercial packaging (batch Nos. NAA6C2B and NAA6NT3), clopidogrel 75-mg and 300-mg tablets were investigator-sourced in commercial packaging (batch Nos. T-01758 and T01924D [300 mg] and 3088070 [75 mg]), and milvexian and matching placebo were provided as 100-mg size 0 gray capsules in 100-count bottles (batch Nos. ABA1625 [milvexian] and ABA2214 [placebo]).

### Ethics

These studies were conducted in accordance with Good Clinical Practice, as defined by the International Council for Harmonisation and in accordance with the ethical principles underlying European Union Directive 2001/20/EC and the United States Code of Federal Regulations and was conducted in accordance with the ethical principles that have their origin in the Declaration of Helsinki. The protocols, amendments, and participant-informed consents received appropriate approval by the Institutional Review Board/Independent Ethics Committee prior to the initiation of the study at the site. Prior to beginning the studies, all participants provided written informed consent.

### Assessments

Safety assessments were performed at selected times throughout the dosing interval (Supplementary Table 7). The assessments were based on medical review of AE and bleeding-related AE reports, and the results of vital sign measurements, ECG measurements, physical examinations, and clinical laboratory tests. PK assessments of milvexian were performed on Days 1 (≤ 24 h) and 5 (≤ 72 h) of each period (Supplementary Table 8); assessments included C_max_, AUC_[(TAU)_ T_max_, and T_1/2_. Individual PK parameters were derived by noncompartmental methods using Phoenix® WinNonlin® PK analysis program (Certara USA, Inc., Princeton, New Jersey; version 8.0). The plasma samples for milvexian, aspirin (acetylsalicylic acid) and its metabolite (salicylic acid), and clopidogrel and its metabolite (clopidogrel acid) were analyzed by validated high-performance liquid chromatography (LC)–tandem mass spectrometry/mass spectrometry (MS/MS) assays^18^. Quantification of milvexian in plasma samples was performed by LC-tandem MS/MS with the quantification range of 1.00 to 1000 ng/mL. Quantitation of clopidogrel and clopidogrel acid in plasma samples were performed by high-performance liquid chromatography (HPLC) coupling MS/MS with the quantification range of 0.200 to 200 ng/mL, and 10.0 to 10,000 ng/mL, respectively. Quantitation of acetylsalicylic acid and salicylic acid in plasma samples were also performed via HPLC coupling MS/MS with the quantification range of 20.0 to 10,000 ng/mL, and 100 to 50,000 ng/mL, respectively.

PD assessments were performed on Days 1 (≤ 24 h) and 5 (≤ 72 h) of each period (Supplementary Table 8); assessments included aPTT^18^, FXIc^18^, and bleeding time (assessed according to the PPD® Laboratories [Austin, TX] procedure where a blood pressure cuff, situated above the incision site, provided pressure at 40 mmHg and blood from the incision was wicked with filter paper every 30 s until cessation to record bleeding time). In addition, AA (1 mM), ADP) (20 µM)-, and collagen (5 μg/ml)-induced platelet aggregation assays were performed using light transmission aggregometry (PPD® Laboratories) and performed on Days 1 and 5 (≤ 24 h) for only Period 1.

### Statistical analyses

All statistical analyses and calculations were performed using SAS® software (SAS Institute, Inc., Cary, North Carolina; version 9.3). All safety and plasma PK/PD data were summarized using descriptive statistics. Descriptive summaries were presented for continuous variables using number of subjects (N), mean, SD; median, minimum, and maximum. GMRs with 90% CIs were calculated to compare effects on plasma PK parameters (C_max_ and AUC_[TAU]_). CIs were constructed using a linear mixed-effects model with treatment as a fixed effect and measurements within participants as repeated measures fitted to the log-transformed PK parameters. These calculations assumed that log transformed PK parameters were normally distributed with intrasubject SD of difference (log scale) ≤ 0.338 and 0.182 for C_max_ and AUC_(TAU)_, respectively. These SDs of difference were estimated from corresponding 90% CI of adjusted GMRs with (1.197, 1.652) and (1.134, 1.348) for acetylsalicylic acid C_max_ and AUC_(TAU)_ observed from a previous study (ClinicalTrials.gov Identifier: NCT03341390) and selected due to higher variabilities compared with the same PK parameters of milvexian and clopidogrel in literature.

### Supplementary Information

Supplementary Information.

## Data Availability

BMS policy on data sharing may be found at https://www.bms.com/researchers-and-partners/independent-research/data-sharing-request-process.html.
