# Supplementary material for: Safety, tolerability, pharmacokinetics and pharmacodynamics of milvexian with aspirin and/or clopidogrel in healthy participants
Source: Sci Rep. 2024 Jul 18;14:16591. doi: 10.1038/s41598-024-67182-8 (PMC11258331; doi:10.1038/s41598-024-67182-8)
Supplement: Supplementary file 1 — Supplementary Information. [file 41598_2024_67182_MOESM1_ESM.docx]

# Supplementary Information

Safety, Tolerability, Pharmacokinetics and Pharmacodynamics of Milvexian With Aspirin and/or Clopidogrel in Healthy Participants

Vidya Perera,^1,^* Grigor Abelian,^1^ Joseph Luettgen,^1^ Ronald Aronson,^1^ Danshi Li,^1^ Zhaoqing Wang,^1^ Liping Zhang,^2^ Susan Lubin,^1^ Samira Merali,^1^ Bindu Murthy^1^

^1^Bristol Myers Squibb, Princeton, NJ, USA; ^2^Janssen Research & Development, LLC, Titusville, NJ, USA.

***Corresponding author.**

3401 Princeton Pike, Lawrenceville, NJ 08648, USA

Phone: 609-302-5464

Email: vid.perera22@gmail.com

# Supplementary Table 1. ALT (U/L) Values for Individual Participants*

| **Participant** | **Day –1** | **Milvexian^†^ +  aspirin**^‡^ **+ clopidogrel**^§^ **(Part 1,  Treatment A)** | **Milvexian^†^  (Part 1,  Treatment B)** | **Aspirin^‡^ + clopidogrel^§^ (Part 1, Treatment C)** | **Participant** | **Day –1** | **Milvexian^†^  (Part 2,  Treatment D)** | **Clopidogrel^§^ (Part 2, Treatment E)** | **Milvexian^†^ + clopidogrel**^§^ **(Part 2,  Treatment F)** | **Participant** | **Day –1** | **Milvexian^†^  (Part 3,  Treatment G)** | **Aspirin^‡^  (Part 3, Treatment H)** | **Milvexian^†^ + aspirin**^‡^ **(Part 3,  Treatment I)** |
| --- | --- | --- | --- | --- | --- | --- | --- | --- | --- | --- | --- | --- | --- | --- |
| **11** | 21 | 17 | 18 | 19 | **116** | 34 |  | 19 |  | **194** | 17 | 11 | 13 |  |
| **15** | 14 | 18 | 21 | 20 | **118** | 20 | 14 | 20 | 15 | **195** | 23 | 24 | 30 | 20 |
| **19** | 22 | 14 | 15 | 16 | **130** | 22 | 26 | 19 | 33 | **196** | 40 | 36 | 34 | 34 |
| **23** | 46 | 17 | 22 | 18 | **131** | 16 | 12 | 8 | 9 | **197** | 9 | 10 | 10 | 10 |
| **25** | 16 | 9 | 8 | 7 | **132** | 9 | 8 | 11 | 9 | **198** | 20 | 22 | 13 | 16 |
| **26** | 30 | 35 |  |  | **140** | 30 | 8 | 8 | 8 | **199** | 21 | 27 | 20 | 22 |
| **28** | 50 | 22 | 24 | 22 | **144** | 19 | 22 | 23 | 23 | **200** | 17 | 16 | 16 | 16 |
| **29** | 27 | 10 | 10 | 12 | **145** | 29 | 18 | 29 | 20 | **211** | 8 | 12 | 10 | 11 |
| **30** | 12 | 7 | 9 | 9 | **147** | 23 | 12 | 14 | 16 | **212** | 19 | 19 | 18 | 19 |
| **32** | 27 | 15 | 17 | 17 | **148** | 35 | 16 | 19 | 14 | **215** | 14 | 9 | 11 | 9 |
| **33** | 13 | 13 | 12 | 11 | **156** | 9 | 12 | 12 | 10 | **217** | 27 | 24 | 29 | 27 |
| **34** | 28 | 21 | 18 | 18 | **159** | 11 | 11 | 10 | 11 | **221** | 14 | 13 | 14 | 17 |
| **41** | 8 | 10 | 10 | 9 | **161** | 14 | 9 | 10 | 9 | **223** | 17 | 12 | 11 | 12 |
| **42** | 21 | 17 | 15 | 21 | **168** | 13 | 11 | 10 | 10 | **224** | 39 | 33 | 40 | 35 |
| **46** | 23 | 37 | 25 | 25 | **174** | 25 | 23 | 25 | 21 | **225** | 29 | 14 | 14 | 12 |
| **63** | 12 | 39 | 36 | 21 | **175** | 38 | 22 | 27 | 31 | **226** | 44 | 17 | 14 | 16 |
| **75** | 47 | 27 | 27 | 22 | **179** | 10 | 10 | 10 | 10 | **228** | 25 | 11 | 14 | 12 |
| **78** | 19 | 16 | 29 | 25 | **181** | 12 | 10 | 11 | 11 | **230** | 24 | 14 | 16 | 17 |
| **90** | 19 | 18 | 19 | 19 | **182** | 30 | 16 | 14 | 14 | **240** | 16 | 9 | 9 | 10 |
| **91** | 16 | 22 | 20 | 20 | **184** | 17 | 13 | 12 | 12 | **247** | 17 | 17 | 21 | 20 |
| **93** | 45 | 29 | 33 | 28 | **185** | 25 | 13 | 16 | 14 | **251** | 12 | 9 | 10 | 8 |
| **96** | 17 | 15 | 14 | 14 | **186** | 14 | 13 | 11 | 12 | **252** | 15 | 17 | 20 | 17 |
| **101** | 12 | 15 | 15 | 31 | **188** | 12 | 12 | 12 | 11 | **254** | 14 | 10 | 13 | 9 |
| **104** | 16 | 9 | 9 | 9 | **189** | 17 | 10 | 9 | 9 | **255** | 21 | 21 | 20 | 23 |
| **106** | 23 | 21 | 24 | 22 | **202** | 13 | 12 | 11 | 12 | **257** | 8 | 11 | 13 | 11 |
| **111** | 27 | 22 | 22 | 19 | **203** | 41 | 21 |  |  | **258** | 14 | 14 | 15 | 16 |
| **113** | 28 | 17 | 19 | 18 | **205** | 10 | 9 | 11 | 7 | **260** | 8 | 7 | 7 | 6 |
| **114** | 27 | 18 | 15 | 15 | **207** | 13 | 9 | 10 | 11 | **262** | 21 | 15 | 15 | 14 |
| **119** | 49 | 25 | 20 | 18 | **208** | 20 | 20 | 21 | 18 | **264** | 15 | 10 | 11 | 10 |
| **120** | 11 | 9 | 10 | 9 | **239** | 18 | 16 | 11 | 11 | **266** | 17 | 13 | 13 | 13 |
| **123** | 18 | 10 | 11 | 9 | **242** | 16 | 11 | 13 | 11 | **269** | 11 |  | 9 |  |
| **127** | 25 | 24 | 22 | 27 | **245** | 19 | 10 | 11 | 11 | **270** | 14 |  |  |  |
| **128** | 14 | 11 | 13 | 15 | **283** | 12 | 10 | 9 | 10 | **271** | 17 | 22 | 22 | 21 |
| **134** | 19 | 10 | 8 | 10 | **285** | 28 | 16 | 17 | 17 | **272** | 12 | 8 | 9 | 8 |
| **138** | 11 | 22 | 10 | 9 | **288** | 12 | 11 | 10 | 10 | **276** | 23 |  |  |  |
| **142** | 10 | 16 | 18 | 16 | **291** | 11 | 10 | 9 | 10 | **282** | 21 | 22 | 19 | 22 |
| **146** | 13 | 12 | 12 | 11 | **293** | 21 | 16 | 15 | 15 | **299** | 37 | 47 | 51 | 40 |
|  |  |  |  |  |  |  |  |  |  | **303** | 51 | 28 | 28 | 29 |
|  |  |  |  |  |  |  |  |  |  | **305** | 17 | 19 | 23 | 20 |

ALT, alanine aminotransferase; BID, twice daily; QD, once daily.

*Normal range = 4-45 for females, 7-56 for males.

^†^Milvexian 200 mg BID on Days 1 to 5.

^‡^Aspirin 325 mg QD on Days 1 to 5.

^§^Clopidogrel 300 mg QD on Day 1 then 75 mg QD on Days 2 to 5.

# Supplementary Table 2. AST (U/L) Values for Individual Participants*

| **Participant** | **Day –1** | **Milvexian^†^ +  aspirin**^‡^ **+ clopidogrel**^§^ **(Part 1,  Treatment A)** | **Milvexian^†^  (Part 1,  Treatment B)** | **Aspirin^‡^ + clopidogrel^§^ (Part 1, Treatment C)** | **Participant** | **Day –1** | **Milvexian^†^  (Part 2,  Treatment D)** | **Clopidogrel^§^ (Part 2, Treatment E)** | **Milvexian^†^ + clopidogrel**^§^ **(Part 2,  Treatment F)** | **Participant** | **Day –1** | **Milvexian^†^  (Part 3,  Treatment G)** | **Aspirin^‡^  (Part 3, Treatment H)** | **Milvexian^†^ + aspirin**^‡^ **(Part 3,  Treatment I)** |
| --- | --- | --- | --- | --- | --- | --- | --- | --- | --- | --- | --- | --- | --- | --- |
| **11** | 19 | 15 | 14 | 16 | **116** | 26 |  | 20 |  | **194** | 18 | 15 | 16 |  |
| **15** | 17 | 15 | 17 | 14 | **118** | 17 | 13 | 15 | 14 | **195** | 21 | 19 | 24 | 20 |
| **19** | 21 | 14 | 15 | 15 | **130** | 23 | 23 | 22 | 30 | **196** | 17 | 17 | 16 | 16 |
| **23** | 22 | 17 | 18 | 18 | **131** | 11 | 10 | 9 | 9 | **197** | 13 | 12 | 12 | 13 |
| **25** | 20 | 11 | 12 | 11 | **132** | 13 | 12 | 13 | 13 | **198** | 19 | 15 | 13 | 14 |
| **26** | 24 | 21 |  |  | **140** | 22 | 13 | 13 | 14 | **199** | 22 | 22 | 18 | 20 |
| **28** | 26 | 18 | 19 | 18 | **144** | 19 | 17 | 17 | 17 | **200** | 18 | 17 | 16 | 16 |
| **29** | 19 | 14 | 14 | 14 | **145** | 30 | 20 | 28 | 22 | **211** | 13 | 14 | 14 | 13 |
| **30** | 13 | 12 | 11 | 12 | **147** | 21 | 13 | 15 | 15 | **212** | 15 | 14 | 13 | 14 |
| **32** | 20 | 16 | 17 | 14 | **148** | 29 | 18 | 17 | 17 | **215** | 19 | 13 | 13 | 12 |
| **33** | 14 | 14 | 12 | 13 | **156** | 12 | 13 | 14 | 14 | **217** | 18 | 15 | 18 | 17 |
| **34** | 21 | 13 | 12 | 12 | **159** | 15 | 14 | 14 | 13 | **221** | 13 | 11 | 11 | 13 |
| **41** | 13 | 11 | 12 | 11 | **161** | 14 | 12 | 13 | 13 | **223** | 18 | 15 | 15 | 15 |
| **42** | 22 | 18 | 16 | 17 | **168** | 9 | 9 | 9 | 9 | **224** | 28 | 22 | 26 | 22 |
| **46** | 16 | 19 | 16 | 17 | **174** | 25 | 23 | 22 | 21 | **225** | 25 | 15 | 15 | 14 |
| **63** | 16 | 26 | 23 | 21 | **175** | 19 | 16 | 18 | 18 | **226** | 19 | 13 | 13 | 14 |
| **75** | 25 | 16 | 16 | 15 | **179** | 12 | 14 | 12 | 14 | **228** | 22 | 22 | 21 | 22 |
| **78** | 23 | 17 | 22 | 22 | **181** | 17 | 16 | 17 | 16 | **230** | 21 | 15 | 15 | 16 |
| **90** | 16 | 17 | 16 | 16 | **182** | 16 | 11 | 9 | 10 | **240** | 19 | 13 | 14 | 14 |
| **91** | 19 | 17 | 16 | 17 | **184** | 18 | 15 | 13 | 14 | **247** | 18 | 18 | 18 | 18 |
| **93** | 21 | 18 | 16 | 16 | **185** | 19 | 15 | 15 | 15 | **251** | 22 | 16 | 18 | 15 |
| **96** | 22 | 16 | 17 | 19 | **186** | 16 | 16 | 15 | 16 | **252** | 17 | 13 | 15 | 13 |
| **101** | 14 | 14 | 19 | 22 | **188** | 18 | 16 | 18 | 17 | **254** | 14 | 13 | 14 | 12 |
| **104** | 19 | 14 | 15 | 14 | **189** | 20 | 12 | 12 | 11 | **255** | 23 | 17 | 18 | 17 |
| **106** | 17 | 15 | 17 | 15 | **202** | 12 | 13 | 13 | 13 | **257** | 14 | 16 | 18 | 17 |
| **111** | 22 | 18 | 17 | 18 | **203** | 22 | 16 |  |  | **258** | 17 | 16 | 17 | 17 |
| **113** | 23 | 14 | 14 | 15 | **205** | 10 | 8 | 10 | 8 | **260** | 14 | 12 | 13 | 10 |
| **114** | 14 | 13 | 11 | 10 | **207** | 13 | 13 | 15 | 12 | **262** | 17 | 14 | 14 | 13 |
| **119** | 24 | 14 | 12 | 11 | **208** | 13 | 14 | 14 | 13 | **264** | 16 | 13 | 14 | 14 |
| **120** | 20 | 18 | 21 | 19 | **239** | 14 | 13 | 11 | 11 | **266** | 16 | 15 | 15 | 15 |
| **123** | 17 | 12 | 14 | 13 | **242** | 19 | 14 | 17 | 15 | **269** | 14 |  | 10 |  |
| **127** | 21 | 17 | 15 | 22 | **245** | 17 | 14 | 15 | 14 | **270** | 16 |  |  |  |
| **128** | 22 | 13 | 16 | 16 | **283** | 15 | 15 | 14 | 15 | **271** | 18 | 21 | 20 | 21 |
| **134** | 17 | 13 | 10 | 12 | **285** | 22 | 16 | 14 | 14 | **272** | 15 | 11 | 12 | 13 |
| **138** | 15 | 50 | 14 | 13 | **288** | 18 | 14 | 14 | 15 | **276** | 27 |  |  |  |
| **142** | 18 | 17 | 19 | 18 | **291** | 15 | 13 | 14 | 15 | **282** | 15 | 16 | 16 | 17 |
| **146** | 19 | 17 | 16 | 15 | **293** | 23 | 20 | 19 | 19 | **299** | 17 | 21 | 23 | 17 |
|  |  |  |  |  |  |  |  |  |  | **303** | 25 | 16 | 17 | 18 |
|  |  |  |  |  |  |  |  |  |  | **305** | 20 | 16 | 19 | 16 |

AST, aspartate aminotransferase; BID, twice daily; QD, once daily.

*Normal range = 10-40.

^†^Milvexian 200 mg BID on Days 1 to 5.

^‡^Aspirin 325 mg QD on Days 1 to 5.

^§^Clopidogrel 300 mg QD on Day 1 then 75 mg QD on Days 2 to 5.

# Supplementary Table 3. Serum Creatinine (mg/dL) Values for Individual Participants*

| **Participant** | **Day –1** | **Milvexian^†^ +  aspirin**^‡^ **+ clopidogrel**^§^ **(Part 1,  Treatment A)** | **Milvexian^†^  (Part 1,  Treatment B)** | **Aspirin^‡^ + clopidogrel^§^ (Part 1, Treatment C)** | **Participant** | **Day –1** | **Milvexian^†^  (Part 2,  Treatment D)** | **Clopidogrel^§^ (Part 2, Treatment E)** | **Milvexian^†^ + clopidogrel**^§^ **(Part 2,  Treatment F)** | **Participant** | **Day –1** | **Milvexian^†^  (Part 3,  Treatment G)** | **Aspirin^‡^  (Part 3, Treatment H)** | **Milvexian^†^ + aspirin**^‡^ **(Part 3,  Treatment I)** |
| --- | --- | --- | --- | --- | --- | --- | --- | --- | --- | --- | --- | --- | --- | --- |
| **11** | 0.86 | 0.87 | 0.82 | 0.84 | **116** | 1.07 |  | 1.08 |  | **194** | 0.97 | 0.95 | 0.99 |  |
| **15** | 1.16 | 1.25 | 1.21 | 1.20 | **118** | 0.94 | 1.08 | 1.04 | 1.04 | **195** | 0.92 | 0.93 | 0.97 | 0.97 |
| **19** | 0.84 | 0.87 | 0.92 | 0.88 | **130** | 0.87 | 0.84 | 0.91 | 0.86 | **196** | 0.85 | 0.92 | 0.85 | 0.91 |
| **23** | 1.00 | 1.07 | 1.05 | 1.07 | **131** | 0.94 | 0.97 | 1.05 | 1.05 | **197** | 0.71 | 0.73 | 0.69 | 0.68 |
| **25** | 0.88 | 0.94 | 0.95 | 0.88 | **132** | 0.94 | 0.88 | 0.89 | 0.89 | **198** | 0.98 | 0.99 | 0.92 | 0.96 |
| **26** | 0.87 | 0.90 |  |  | **140** | 0.88 | 0.92 | 0.97 | 1.02 | **199** | 0.99 | 1.09 | 1.00 | 1.00 |
| **28** | 1.04 | 1.15 | 1.13 | 1.10 | **144** | 1.03 | 1.08 | 1.15 | 1.11 | **200** | 0.95 | 1.10 | 1.18 | 1.09 |
| **29** | 1.03 | 1.14 | 1.10 | 1.14 | **145** | 1.02 | 0.96 | 1.05 | 0.92 | **211** | 0.70 | 0.75 | 0.72 | 0.73 |
| **30** | 0.79 | 0.94 | 0.88 | 0.91 | **147** | 1.05 | 1.15 | 1.15 | 1.16 | **212** | 0.91 | 0.91 | 1.00 | 0.98 |
| **32** | 0.90 | 1.02 | 1.03 | 1.11 | **148** | 1.14 | 1.25 | 1.24 | 1.29 | **215** | 0.83 | 0.99 | 0.92 | 0.93 |
| **33** | 0.81 | 0.88 | 0.84 | 0.83 | **156** | 0.97 | 1.16 | 1.06 | 1.11 | **217** | 0.85 | 0.84 | 0.86 | 0.90 |
| **34** | 0.90 | 0.97 | 0.99 | 0.96 | **159** | 1.05 | 1.12 | 1.11 | 1.12 | **221** | 0.88 | 0.98 | 1.01 | 1.05 |
| **41** | 0.97 | 1.01 | 1.00 | 0.99 | **161** | 0.94 | 0.95 | 0.98 | 0.93 | **223** | 1.04 | 1.07 | 1.08 | 1.10 |
| **42** | 0.88 | 0.98 | 1.04 | 0.99 | **168** | 1.06 | 1.12 | 1.09 | 1.12 | **224** | 1.30 | 1.36 | 1.29 | 1.27 |
| **46** | 1.01 | 0.99 | 1.10 | 1.03 | **174** | 1.17 | 1.16 | 1.14 | 1.22 | **225** | 0.91 | 0.83 | 0.81 | 0.91 |
| **63** | 0.99 | 0.94 | 0.84 | 0.85 | **175** | 1.13 | 1.30 | 1.23 | 1.29 | **226** | 0.76 | 0.88 | 0.91 | 0.87 |
| **75** | 0.91 | 1.02 | 1.04 | 1.04 | **179** | 0.92 | 1.01 | 1.02 | 1.02 | **228** | 1.09 | 1.03 | 1.05 | 1.08 |
| **78** | 1.03 | 1.09 | 1.04 | 1.06 | **181** | 0.74 | 0.76 | 0.73 | 0.72 | **230** | 0.95 | 0.92 | 0.95 | 0.93 |
| **90** | 1.05 | 1.04 | 1.03 | 1.05 | **182** | 0.77 | 0.87 | 0.79 | 0.90 | **240** | 0.78 | 0.83 | 0.78 | 0.80 |
| **91** | 0.80 | 1.00 | 0.94 | 0.90 | **184** | 0.71 | 0.78 | 0.79 | 0.75 | **247** | 0.85 | 0.81 | 0.81 | 0.80 |
| **93** | 1.20 | 1.09 | 1.12 | 1.12 | **185** | 0.75 | 0.75 | 0.78 | 0.81 | **251** | 1.06 | 1.20 | 1.14 | 1.15 |
| **96** | 1.06 | 1.06 | 1.05 | 1.04 | **186** | 0.87 | 0.99 | 0.92 | 0.95 | **252** | 0.97 | 1.01 | 0.99 | 1.04 |
| **101** | 0.88 | 0.92 | 0.89 | 0.86 | **188** | 0.79 | 0.85 | 0.92 | 0.79 | **254** | 0.94 | 0.97 | 0.90 | 0.95 |
| **104** | 0.97 | 0.99 | 0.99 | 0.99 | **189** | 0.79 | 0.88 | 0.89 | 0.89 | **255** | 1.00 | 1.16 | 1.14 | 1.13 |
| **106** | 0.84 | 0.93 | 0.92 | 1.06 | **202** | 1.01 | 1.02 | 0.96 | 0.95 | **257** | 1.05 | 1.12 | 1.12 | 1.03 |
| **111** | 0.75 | 0.89 | 0.84 | 0.85 | **203** | 1.01 | 0.99 |  |  | **258** | 1.04 | 1.05 | 1.06 | 1.11 |
| **113** | 1.01 | 1.14 | 1.05 | 1.07 | **205** | 0.92 | 1.05 | 1.03 | 1.09 | **260** | 1.01 | 1.08 | 1.15 | 1.07 |
| **114** | 0.89 | 0.84 | 0.84 | 0.84 | **207** | 1.03 | 0.96 | 1.07 | 1.02 | **262** | 0.61 | 0.66 | 0.70 | 0.63 |
| **119** | 1.10 | 1.14 | 1.14 | 1.11 | **208** | 1.19 | 1.20 | 1.23 | 1.26 | **264** | 1.03 | 1.30 | 1.16 | 1.23 |
| **120** | 0.84 | 0.89 | 0.88 | 0.89 | **239** | 1.03 | 1.03 | 1.11 | 1.03 | **266** | 0.96 | 1.03 | 1.03 | 1.04 |
| **123** | 0.97 | 0.98 | 1.02 | 0.93 | **242** | 1.36 | 1.29 | 1.20 | 1.30 | **269** | 0.81 |  | 0.86 |  |
| **127** | 1.14 | 1.24 | 1.29 | 1.31 | **245** | 1.11 | 1.16 | 1.21 | 1.15 | **270** | 1.06 |  |  |  |
| **128** | 0.92 | 1.06 | 1.10 | 1.00 | **283** | 1.22 | 1.18 | 1.16 | 1.11 | **271** | 1.08 | 1.18 | 1.12 | 1.10 |
| **134** | 0.74 | 0.86 | 0.80 | 0.81 | **285** | 1.07 | 1.17 | 1.20 | 1.18 | **272** | 0.99 | 1.00 | 1.02 | 1.02 |
| **138** | 1.04 | 1.08 | 1.03 | 1.07 | **288** | 1.15 | 1.12 | 1.10 | 1.18 | **276** | 0.98 |  |  |  |
| **142** | 1.29 | 1.25 | 1.14 | 1.10 | **291** | 1.07 | 1.12 | 1.12 | 1.10 | **282** | 0.99 | 0.99 | 1.03 | 1.01 |
| **146** | 1.28 | 1.34 | 1.35 | 1.36 | **293** | 0.83 | 0.97 | 0.88 | 0.89 | **299** | 0.89 | 0.88 | 0.85 | 0.90 |
|  |  |  |  |  |  |  |  |  |  | **303** | 0.75 | 0.80 | 0.75 | 0.76 |
|  |  |  |  |  |  |  |  |  |  | **305** | 0.96 | 1.12 | 1.13 | 1.10 |

ALT, alanine aminotransferase; AST, aspartate aminotransferase; BID, twice daily; QD, once daily.

*Normal range = 0.45-1.06 for females, 0.64-1.30 for males.

^†^Milvexian 200 mg BID on Days 1 to 5.

^‡^Aspirin 325 mg QD on Days 1 to 5.

^§^Clopidogrel 300 mg QD on Day 1 then 75 mg QD on Days 2 to 5.

# Supplementary Table 4. PK Parameters of Milvexian on Days 1 and 5

|  | **Milvexian** | | | | | |
| --- | --- | --- | --- | --- | --- | --- |
| **PK parameter** | **Milvexian^*^ +  aspirin**^†^ **+ clopidogrel**^‡^ **(Part 1,  Treatment A)**  **(n = 37)** | **Milvexian^*^ + clopidogrel**^‡^ **(Part 2,  Treatment F)**  **(n = 35)** | **Milvexian^*^ + aspirin**^†^ **(Part 3,  Treatment I)**  **(n = 35)** | **Milvexian^*^  (Part 1,  Treatment B)**  **(n = 36)** | **Milvexian^*^  (Part 2,  Treatment D)**  **(n = 36)** | **Milvexian^*^  (Part 3,  Treatment G)**  **(n = 37)** |
| **Day 1** |  |  |  |  |  |  |
| C_max_, ng/mL, geometric mean (90% CI) | 718 (632, 815) | 817 (746, 895) | 850 (753, 959) | 868 (769, 980) | 898 (821, 981) | 867 (793, 947) |
| AUC_(TAU)_, ng•h/mL, geometric mean [n]^§^ (90% CI) | 4773 [36]  (4248, 5363) | 5042  (4621, 5503) | 5553 [32]  (4985, 6185) | 5610  (5005, 6288) | 5648 [33]  (5125, 6225) | 5609 [35]  (5115, 6151) |
| T_max_, h, median (range) | 4.00 (2.13, 6.02) | 4.00 (2.00, 4.13) | 4.00 (2.00, 6.03) | 3.00 (2.00, 4.13) | 4.00 (2.00, 6.00) | 4.00 (2.00, 6.02) |
| T_1/2_, h, arithmetic mean [n]^§^ (SD) | 4.21 [6] (0.277) | 3.83 [8] (0.306) | 4.07 [8] (0.583) | 3.89 [9] (0.346) | 4.13 [8] (0.303) | 3.90 [9] (0.400) |
| **Day 5** |  |  |  |  |  |  |
| C_max_, ng/mL, geometric mean [n]^§^ (90% CI) | 2414 [36]  (2241, 2599) | 2498  (2288, 2728) | 2654 [34]  (2411, 2923) | 2689  (2496, 2897) | 2672  (2511, 2843) | 2547 [36]  (2359, 2749) |
| AUC_(TAU)_, ng•h/mL, geometric mean [n]^§^ (90% CI) | 20,859 [35]  (19,239, 22,617) | 19,851 [33]  (18,048, 21,833) | 22,216 [32]  (20,221, 24,408) | 22,216 [35]  (20,598, 23,961) | 22,018  (20,472, 23,681) | 21,956 [31]  (20,021, 24,079) |
| T_max_, h, median [n]^§^ (range) | 4.00 [36] (1.00, 6.00) | 4.00 (1.00, 6.00) | 4.00 [34] (1.00, 11.9) | 3.00 (0.00, 6.00) | 3.05 (2.00, 4.05) | 3.00 [36] (0.500, 6.02) |
| T_1/2_, h, arithmetic mean [n]^§^ (SD) | 12.2 [36] (2.46) | 11.5 (2.28) | 13.1 [33] (4.03) | 11.4 [32] (1.99) | 11.2 [35] (1.94) | 13.1 [35] (3.87) |

AUC_(TAU)_, area under the plasma concentration-time curve from time 0 to time of last quantifiable concentration; BID, twice daily; CI, confidence interval; C_max_, maximum observed concentration; PK, pharmacokinetic; QD, once daily; SD, standard deviation; T_max_, time of maximum observed concentration; T_1/2_, terminal plasma half-life.

*Milvexian 200 mg BID on Days 1 to 5.

^†^Aspirin 325 mg QD on Days 1 to 5.

^‡^Clopidogrel 300 mg QD on Day 1 then 75 mg QD on Days 2 to 5.

^§^Number of participants included in the analysis if different from the overall group size.

# Supplementary Table 5. PK Parameter Summaries of Clopidogrel and Its Metabolite (Clopidogrel Acid) on Days 1 and 5

|  | **Clopidogrel** | | | | **Clopidogrel acid** | | | |
| --- | --- | --- | --- | --- | --- | --- | --- | --- |
| **PK parameter** | **Milvexian^*^ + aspirin**^†^ **+ clopidogrel**^‡^ **(Part 1, Treatment A)**  **(n = 37)** | **Placebo + aspirin**^†^ **+ clopidogrel**^‡^ **(Part 1, Treatment C)**  **(n = 36)** | **Milvexian^*^ + clopidogrel**^‡^ **(Part 2, Treatment F)**  **(n = 35)** | **Placebo + clopidogrel**^‡^ **(Part 2, Treatment E)**  **(n = 36)** | **Milvexian^*^ + aspirin**^†^ **+ clopidogrel**^‡^ **(Part 1, Treatment A)**  **(n = 37)** | **Placebo + aspirin**^†^ **+ clopidogrel**^‡^ **(Part 1, Treatment C)**  **(n = 36)** | **Milvexian^*^ + clopidogrel**^‡^ **(Part 2, Treatment F)**  **(n = 35)** | **Placebo + clopidogrel**^‡^ **(Part 2, Treatment E)**  **(n = 36)** |
| **Day 1** |  |  |  |  |  |  |  |  |
| C_max_, ng/mL, geometric mean (90% CI) | 3.29  (2.58, 4.21) | 3.83  (3.08, 4.76) | 4.05  (3.09, 5.31) | 4.45  (3.40, 5.82) | 10,451  (9429, 11,584) | 11,955  (10,963, 13,035) | 11,569  (10,726, 12,478) | 1174  (10,875, 12,674) |
| AUC_(TAU)_, ng•h/mL, geometric mean [n]^§^ (90% CI) | 8.75 [32]  (7.04, 10.9) | 8.66 [30]  (7.32, 10.2) | 8.76 [32]  (6.73, 11.4) | 9.44 [33]  (7.32, 12.2) | 39,092  (36,516, 41,850) | 41,932  (39,070, 45,003) | 43,047  (39,600, 46,794) | 43,292  (39,851, 47,031) |
| T_max_, h, median (range) | 1.00 (0.500, 6.02) | 1.00 (0.500, 5.05) | 1.50 (0.500, 3.00) | 1.00 (0.500, 3.00) | 1.00 (0.500, 3.00) | 1.00 (0.500, 2.07) | 1.50 (1.00, 3.00) | 1.00 (0.500, 3.00) |
| T_1/2_, h, arithmetic mean [n]^§^ (SD) | 2.10 [28] (1.607) | 1.74 [26] (1.486) | 2.42 [29] (2.452) | 2.00 [32] (1.816) | 9.69 [35] (4.274) | 8.10 (2.449) | 8.20 (1.694) | 8.02 (2.562) |
| **Day 5** |  |  |  |  |  |  |  |  |
| C_max_, ng/mL, geometric mean [n]^§^ (90% CI) | 1.04 [36]  (0.827, 1.30) | 1.15  (0.898, 1.46) | 1.26  (0.954, 1.66) | 1.37 [35]  (1.03, 1.84) | 3248 [36]  (2958, 3566) | 3333  (2995, 3709) | 3234  (2940, 3557) | 3738 [35]  (3377, 4137) |
| AUC_(TAU)_, ng•h/mL, geometric mean [n]^§^ (90% CI) | 3.08 [16]  (2.44, 3.89) | 2.83 [18]  (2.34, 3.43) | 3.16 [19]  (2.30, 4.33) | 3.25 [16]  (2.42, 4.36) | 9574 [36]  (8923, 10,271) | 9472  (8829, 10,162) | 10,347  (9454, 11,325) | 9982 [35]  (9101, 10,948) |
| T_max_, h, median [n]^§^ (range) | 1.00 [36]  (0.000, 2.00) | 0.500  (0.500, 5.00) | 1.00  (0.000, 2.00) | 0.500 [35] (0.500, 1.50) | 0.508 [36] (0.500, 1.50) | 0.500  (0.500, 2.00) | 0.500  (0.500, 3.00) | 0.500 [35] (0.500, 1.50) |
| T_1/2_, h, arithmetic mean [n]^§^ (SD) | 0.753 [9] (0.2659) | 0.751 [15] (0.2661) | 1.10 [16]  (1.122) | 1.07 [12]  (1.138) | 8.58 [36]  (2.377) | 8.19  (2.552) | 8.35  (1.892) | 8.59 [35]  (2.347) |

AUC _(TAU)_, area under the plasma concentration-time curve from time 0 to time of last quantifiable concentration; BID, twice daily; CI, confidence interval; C_max_, maximum observed concentration; PK, pharmacokinetic; QD, once daily; SD, standard deviation; T_max_, time of maximum observed concentration; T_1/2_, terminal plasma half-life.

^*^Milvexian 200 mg BID on Days 1 to 5.

^†^Aspirin 325 mg QD on Days 1 to 5.

^‡^Clopidogrel 300 mg QD on Day 1 then 75 mg QD on Days 2 to 5.

^§^Number of participants included in the analysis if different from the overall group size.

# Supplementary Table 6. PK Parameter Summaries of Aspirin (Acetylsalicylic Acid) and Its Metabolite (Salicylic Acid on Days 1 and 5

|  | **Aspirin (acetylsalicylic acid)** | | | | **Salicylic acid** | | | |
| --- | --- | --- | --- | --- | --- | --- | --- | --- |
| **PK parameter** | **Milvexian^*^ + aspirin**^†^ **+ clopidogrel**^‡^ **(Part 1, Treatment A)**  **(n = 37)** | **Placebo + aspirin**^†^ **+ clopidogrel**^‡^ **(Part 1, Treatment C)**  **(n = 36)** | **Milvexian^*^ + aspirin**^†^  **(Part 3, Treatment I)**  **(n = 35)** | **Placebo + aspirin**^†^ **(Part 3, Treatment H)**  **(n = 38)** | **Milvexian^*^ + aspirin**^†^ **+ clopidogrel**^‡^ **(Part 1, Treatment A)**  **(n = 37)** | **Placebo + aspirin**^†^ **+ clopidogrel**^‡^ **(Part 1, Treatment C)**  **(n = 36)** | **Milvexian^*^ + aspirin**^†^ **(Part 3, Treatment I)**  **(n = 35)** | **Placebo + aspirin**^†^ **(Part 3, Treatment H)**  **(n = 38)** |
| **Day 1** |  |  |  |  |  |  |  |  |
| C_max_, ng/mL, geometric mean (90% CI) | 3049  (2739, 3394) | 2967  (2638, 3337) | 3445  (3060, 3879) | 2454  (2219, 2714) | 17,641  (16,753, 18,576) | 18,107  (17,232, 19,026) | 16,294  (15,371, 17,272) | 16,482  (15,595, 17,419) |
| AUC_(TAU)_, ng•h/mL, geometric mean [n]^§^ (90% CI) | 4967 [35]  (4687, 5263) | 4460 [31]  (4126, 4822) | 4674 [34]  (4425, 4937) | 3769 [36]  (3574, 3975) | 92,737  (86,976, 98,880) | 93,483  (87,900, 99,420) | 93,633  (86,856, 100,938) | 93,270  (85,997, 101,158) |
| T_max_, h, median (range) | 1.00  (0.500, 2.00) | 1.00  (0.250, 2.07) | 0.500  (0.250, 2.00) | 0.500  (0.250, 3.00) | 2.00  (1.50, 4.03) | 2.00  (1.00, 5.05) | 2.00  (1.00, 5.00) | 2.03  (1.00, 5.00) |
| T_1/2_, h, arithmetic mean [n]^§^ (SD) | 0.506 [35] (0.3984) | 0.503 [31] (0.2665) | 0.487 [34] (0.1463) | 0.497 [36] (0.1293) | 2.21  (0.393) | 2.25  (0.479) | 2.39  (0.538) | 2.40  (0.656) |
| **Day 5** |  |  |  |  |  |  |  |  |
| C_max_, ng/mL, geometric mean [n]^§^ (90% CI) | 3234 [36]  (2820, 3709) | 2893  (2573, 3253) | 3557 [33]  (3049, 4150) | 2670  (2347, 3037) | 16,882 [36] (15,693, 18,162) | 17,299  (16,286, 18,375) | 16,349 [34] (15,277, 17,496) | 16,494  (15,441, 17,619) |
| AUC_(TAU)_, ng•h/mL, geometric mean [n]^§^ (90% CI) | 5376 [34]  (5012, 5766) | 4415 [33]  (4078, 4778) | 4972 [33]  (4699, 5262) | 4163 [31]  (3923, 4419) | 92,254 [36] (86,332, 98,583) | 91,987  (86,331, 98,015) | 94,200 [34] (87,163, 101,805) | 92,528  (85,636, 99,975) |
| T_max_, h, median [n]^§^ (range) | 0.500 [36] (0.250, 3.00) | 0.500  (0.500, 3.00) | 0.500 [34] (0.250, 3.00) | 0.508  (0.250, 4.02) | 2.07 [36]  (1.50, 5.00) | 2.02  (1.00, 5.00) | 2.00 [34]  (1.00, 5.00) | 3.00  (1.00, 5.00) |
| T_1/2_, h, arithmetic mean [n]^§^ (SD) | 0.468 [30] (0.0930) | 0.481 [28] (0.3042) | 0.463 [28] (0.0918) | 0.487 [27] (0.1342) | 2.25 [36]  (0.416) | 2.23  (0.423) | 2.41 [33]  (0.598) | 2.39  (0.565) |

AUC_(Tau)_, area under the plasma concentration-time curve from time 0 to time of last quantifiable concentration; BID, twice daily; CI, confidence interval; C_max_, maximum observed concentration; PK, pharmacokinetic; QD, once daily; SD, standard deviation; T_max_, time of maximum observed concentration; T_1/2_, terminal plasma half-life.

^*^Milvexian 200 mg BID on Days 1 to 5.

^†^Aspirin 325 mg QD on Days 1 to 5.

^‡^Clopidogrel 300 mg QD on Day 1 then 75 mg QD on Days 2 to 5.

^§^Number of participants included in the analysis if different from the overall group size.

# Supplementary Table 7. Safety Assessment Schedule (Parts 1-3)

| **Safety assessment** | **Baseline (day)** | **Period 1 (day)** | | | | | | **Period 2 (day)** | | | | | | **Period 3 (day)** | | | | | | | **Study discharge** |
| --- | --- | --- | --- | --- | --- | --- | --- | --- | --- | --- | --- | --- | --- | --- | --- | --- | --- | --- | --- | --- | --- |
|  | –1 | 1 | 2 | 3 | 4 | 5 | W^*^ | 1 | 2 | 3 | 4 | 5 | W^*^ | 1 | 2 | 3 | 4 | 5 | 6 | 7 | 8 |
| Physical examination | X |  |  |  |  |  | X |  |  |  |  |  | X |  |  |  |  |  |  | X^†^ |  |
| Physical measurements | X |  |  |  |  |  |  |  |  |  |  |  |  |  |  |  |  |  |  | X^†^ |  |
| Vital signs | X |  |  |  |  | X |  | X |  |  |  | X |  | X |  |  |  | X |  |  | X |
| Electrocardiogram | X |  |  |  |  | X | X |  |  |  |  | X | X |  |  |  |  | X |  | X^†^ |  |
| Clinical laboratory tests | X |  |  |  |  |  | X |  |  |  |  |  | X |  |  |  |  |  |  | X^†^ |  |
| Hemoglobin and hematocrit | X |  |  | X |  | X |  |  |  | X |  | X |  |  |  | X |  | X |  |  |  |
| Concomitant medication use | X | X | X | X | X | X | X | X | X | X | X | X | X | X | X | X | X | X | X | X | X |
| Serious AE monitoring | X | X | X | X | X | X | X | X | X | X | X | X | X | X | X | X | X | X | X | X | X |
| Nonserious AE monitoring |  | X | X | X | X | X | X | X | X | X | X | X | X | X | X | X | X | X | X | X | X |

AE, adverse event; W, washout.

^*^Seven-day washout between last dose of study drug in current period and first dose in subsequent period.

^†^Evaluations performed prior to study discharge, or when participants were prematurely discontinued.

# Supplementary Table 8. PK and PD Sampling Schedule (Parts 1-3)

| **Study day** | **Time**  **(relative to milvexian dose), hour:min** | **Milvexian blood sample for PK** | **Acetylsalicylic acid and salicylic acid blood sample for PK** | **Clopidogrel and clopidogrel metabolite blood sample for PK** | **PD blood sample for platelet aggregation (Period 1 only)** | **PD blood sample for bleeding time** | **PD blood sample for aPTT and FXIc** | **PD blood sample for thrombin time** |
| --- | --- | --- | --- | --- | --- | --- | --- | --- |
| 1 | 00:00 (predose) | X^*^ | X | X | X | X | X | X |
| 1 | 00:15 |  | X | X |  |  |  |  |
| 1 | 00:30 | X | X | X |  |  |  |  |
| 1 | 1:00 | X | X | X |  |  |  |  |
| 1 | 1:30 |  | X | X |  |  |  |  |
| 1 | 2:00 | X | X | X | X |  | X | X |
| 1 | 3:00 | X | X | X |  |  |  |  |
| 1 | 4:00 | X | X | X | X | X | X | X |
| 1 | 5:00 |  | X | X |  |  |  |  |
| 1 | 6:00 | X | X | X |  |  |  |  |
| 1 | 8:00 | X | X | X |  |  | X |  |
| 1 | 12:00 | X^*^ | X | X |  |  | X |  |
| 1 | 16:00 |  | X | X |  |  | X |  |
| 2 | 00:00 (predose) | X | X | X | X |  | X | X |
| 3 | 00:00 (predose) | X | X | X |  |  |  |  |
| 4 | 00:00 (predose) | X | X | X |  |  |  |  |
| 5 | 00:00 (predose) | X^*^ | X | X | X | X | X | X |
| 5 | 00:15 |  | X | X |  |  |  |  |
| 5 | 00:30 | X | X | X |  |  |  |  |
| 5 | 1:00 |  | X | X |  |  |  |  |
| 5 | 1:30 |  | X | X |  |  |  |  |
| 5 | 2:00 | X | X | X | X |  | X | X |
| 5 | 3:00 | X | X | X |  |  |  |  |
| 5 | 4:00 | X | X | X | X | X | X | X |
| 5 | 5:00 |  | X | X |  |  |  |  |
| 5 | 6:00 | X | X | X |  |  |  |  |
| 5 | 8:00 | X | X | X |  |  | X |  |
| 5 | 12:00 | X^*^ | X | X |  |  | X |  |
| 5 | 16:00 | X | X | X |  |  | X |  |
| 6 | 24:00 | X | X | X | X |  | X |  |
| 6 | 36:00 | X |  | X |  |  | X |  |
| 7 | 48:00 | X |  | X |  |  | X |  |
| 8 | 72:00 | X |  | X |  |  | X | X |

aPTT, activated partial thromboplastin time; FXIc, Factor XI clotting activity; PD, pharmacodynamic; PK, pharmacokinetic.

*Sample obtained prior to milvexian administration.

# Supplementary Figure 1. Mean (± SD) milvexian plasma concentration versus time profile of milvexian control groups on Days 1 and 5.^*^

**
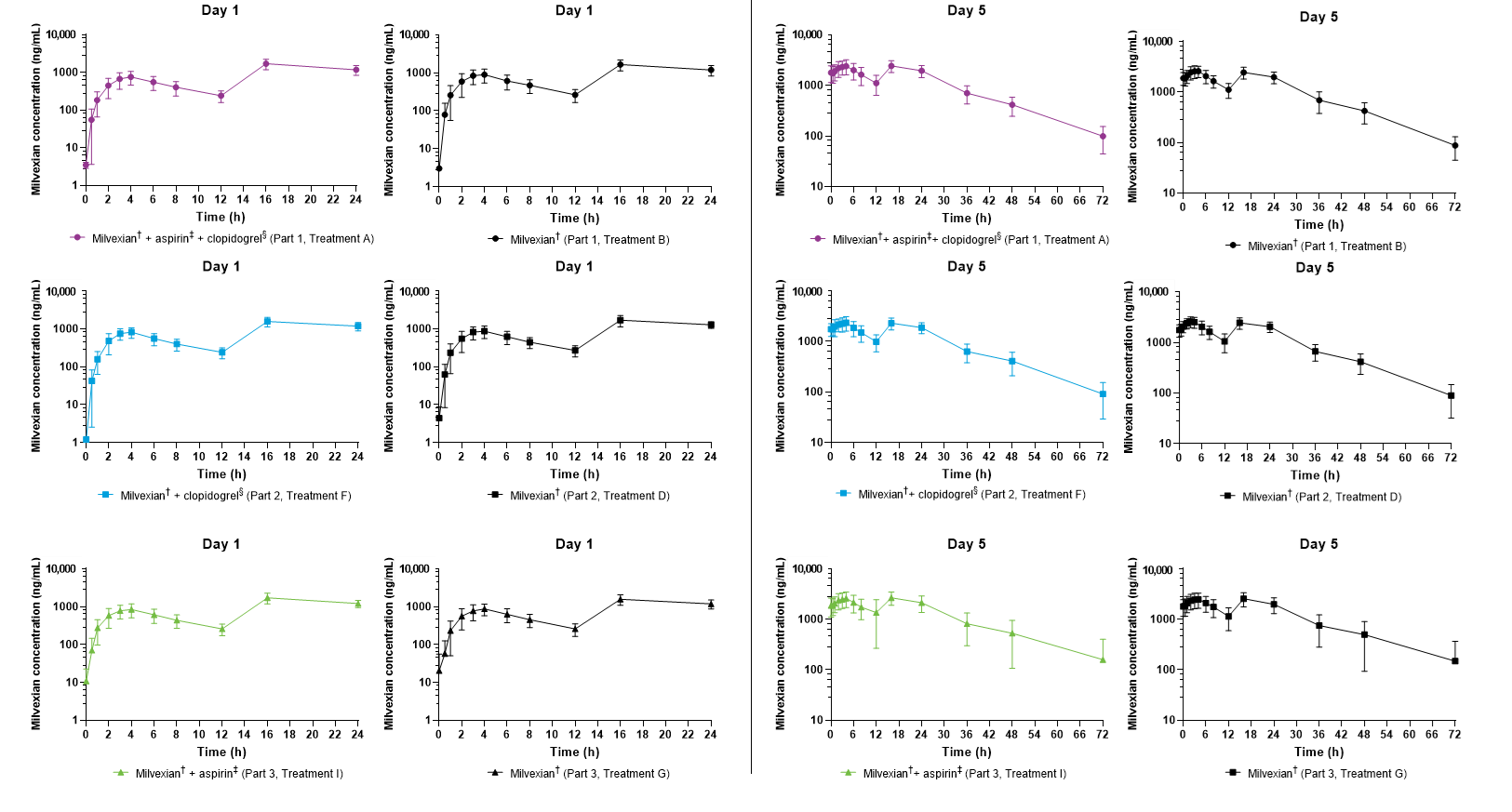
**

BID, twice daily; QD, once daily; SD, standard deviation.

*Overlay plots of the curves are reported in Figure 2.

^†^Milvexian 200 mg BID on Days 1 to 5.

^‡^Aspirin 325 mg QD on Days 1 to 5.

^§^Clopidogrel 300 mg QD on Day 1 then 75 mg QD on Days 2 to 5.
